# Exosomal miRNA profile as complementary tool in the diagnostic and prediction of treatment response in localized breast cancer under neoadjuvant chemotherapy

**DOI:** 10.1186/s13058-019-1109-0

**Published:** 2019-02-06

**Authors:** Alba Rodríguez-Martínez, Diego de Miguel-Pérez, Francisco Gabriel Ortega, José Luis García-Puche, Inmaculada Robles-Fernández, José Exposito, Jordi Martorell-Marugan, Pedro Carmona-Sáez, María del Carmen Garrido-Navas, Christian Rolfo, Hugh Ilyine, José Antonio Lorente, Marta Legueren, María José Serrano

**Affiliations:** 1 Liquid biopsy and metastasis research group, GENYO, Centre for Genomics and Oncological Research, Pfizer/University of Granada/Andalusian Regional Government PTS, Granada, Avenida de la Ilustración 114, 18016 Granada, Spain; 2 Laboratory of Genetic Identification, Legal Medicine and Toxicology Department, Faculty of Medicine, University of Granada, Avenida de la Investigación, 11, 18071 Granada, Spain; 3 Comprehensive oncology division, Clinical University Hospital, Virgen de las Nieves-San Cecilio, Av. de las Fuerzas Armadas, 2, 18014 Granada, Spain; 4 Bioinformatics Unit, GENYO, Centre for Genomics and Oncological Research: Pfizer/University of Granada/Andalusian Regional Government PTS. Granada, Avenida de la Ilustración, 114, 18016 Granada, Spain; 5 Thoracic Medical Oncology, Early Clinical Trials, University of Maryland Marlene and Stewart Greenebaum Comprehensive Cancer Center (UMGCCC), 22 S. Greene Street, Baltimore, 21201 USA; 6 DestiNA Genomics Ltd, 7-11 Melville St, Edinburgh, EH3 7PE UK

**Keywords:** Exosomes, microRNA, Circulating tumor cells, Localized breast cancer, Neoadjuvant chemotherapy, Conservative surgery, Cancer diagnosis, Cancer prognosis

## Abstract

**Background:**

Breast cancer patients under neoadjuvant chemotherapy includes a heterogeneous group of patients who eventually develop distal disease, not detectable by current methods. We propose the use of exosomal miRNAs and circulating tumor cells as diagnostic and predictive biomarkers in these patients.

**Methods:**

Fifty-three breast cancer women initially diagnosed with localized breast cancer under neoadjuvant chemotherapy were prospectively enrolled in this study. However, six of them were later re-evaluated and diagnosed as metastatic breast cancer patients by PET-CT scan. Additionally, eight healthy donors were included. Circulating tumor cells and serum exosomal miRNAs were isolated from blood samples before and at the middle of neoadjuvant therapy and exosomal miRNA levels analyzed by qPCR.

**Results:**

Before neoadjuvant therapy, exosomal miRNA-21 and 105 expression levels were higher in metastatic versus non-metastatic patients and healthy donors. Likewise, higher levels of miRNA-222 were observed in basal-like (*p* = 0.037) and in luminal B versus luminal A (*p* = 0.0145) tumor subtypes. Exosomal miRNA-222 levels correlated with clinical and pathological variables such as progesterone receptor status (*p* = 0.017) and Ki67 (*p* = 0.05). During neoadjuvant treatment, exosomal miRNA-21 expression levels directly correlated with tumor size (*p* = 0.039) and inversely with Ki67 expression (*p* = 0.031). Finally, higher levels of exosomal miRNA-21, miRNA-222, and miRNA-155 were significantly associated with the presence of circulating tumor cells.

**Conclusion:**

Liquid biopsies based on exosomal miRNAs and circulating tumor cells can be a complementary clinical tool for improving breast cancer diagnosis and prognosis.

**Electronic supplementary material:**

The online version of this article (10.1186/s13058-019-1109-0) contains supplementary material, which is available to authorized users.

## Background

Breast cancer (BC) is the most frequently diagnosed cancer in women and the second most common cancer worldwide [1]*.* Despite considerable advances in early detection, diagnosis, and treatment, BC is among the leading causes of cancer-related deaths in women due to recurrent metastatic disease [2]*.* Approximately 20 to 25% of women diagnosed with localized BC (LBC) are subjected to neoadjuvant therapy [3]. These tumors remain a noteworthy clinical problem, as a significative percentage of these patients will develop metastatic disease, despite appropriate treatment [4, 5]*.* Therefore, early detection of the systemic disease is especially important for improving the clinical outcomes in these patients [6]*.* Thus, despite the improvement of the imaging techniques and diagnostic biomarkers, they are not yet fully satisfactory, principally due to important limitations in detecting distal disease [7, 8]*.* In this context, liquid biopsy (LB) emerges as an increasing important tool for early tumor diagnosis, recurrence monitoring, and therapeutic guidance [9]*.* LB provides a non-invasive alternative to traditional “solid biopsies,” which cannot be consistently performed in certain situations, in “real time,” or as easily under recurring sampling and monitoring. LB also has significant value improving our knowledge about the metastatic processes occurring in blood. Consequently, with further clinical validation, LB could allow a better patient risk stratification and, therefore, a better patient treatment choice [10]*.*

The presence of circulating tumor cells (CTCs) and cell-free nucleic acids (cfNAs) such as DNA, mRNA, and microRNA in blood has been recognized, and their clinical relevance is considered attractive as novel biomarkers [11, 12]*.*

CTCs can be used as markers of disease progression, as early indicators of metastasis, and as mediators of drug resistance in BC [13, 14].

The interest of exosomal microRNAs (EmiRs) in cancer has been vastly intensified [15]. In fact, aberrantly expressed microRNAs in tissues, serum/plasma, and CTCs have been explored in the development of new BC biomarkers [16–18]. However, this has not yet been proved to be successful, mainly due to confounding factors impacting levels of circulating miRNAs and potentially compromising their potential as disease biomarkers [19]. In response to this, exosomes are suggested, as containers of miRNAs, to solve these problems [20].

In this work, we sought to evaluate the role of an EmiR panel in the diagnosis and prediction of treatment response in BC patients under neoadjuvant therapy. We further correlated the CTC findings with specific EmiR profiles and clinical and pathological characteristics of the matching primary tumors.

## Methods

### Experimental design

BC women (*n* = 53) initially diagnosed with LBC susceptible to receive neoadjuvant chemotherapy were prospectively enrolled in this study. Forty-seven of them underwent neoadjuvant treatment while the other six, initially diagnosed as LBC, were newly evaluated and considered as metastatic breast cancer (MBC) after PET-CT scan evaluation (Additional file 1). Peripheral blood samples (10 ml in EDTA Vacutainer® tubes for CTCs and 5 ml in BD Vacutainer® SST™ II Advance tubes for serum) were extracted at diagnosis time (Ext1) and after 4 cycles of doxorubicin/cyclophosphamide (Ext2). CTCs and exosomes were isolated following the protocol established by our group [10], and identification and counting were performed in a computerized fluorescence microscope (Zeiss AXIO Imager) (Additional file 1).

### Biomarker analysis

miRNAs were extracted from exosomes using the Maxwell® 16 miRNA Tissue kit (Promega, USA). Complementary DNA was synthesized using 10 ng of total miRNA and the TaqMan™ Advanced miRNA cDNA Synthesis Kit (Applied Biosystems, USA) following the manufacturer’s protocol. A panel of five miRNAs was designed based on their association with previously reported regulatory roles in cell proliferation, dissemination, and invasion when overexpressed in BC. In addition, the selection was based on their reported mRNA targets of genes as ESR1, progesterone receptors (PGR), and ERBB2 and oncogenes as FOXO3, using the database starBase v3.0 [21]: miR21-5p (significantly correlated with advanced clinical stage, lymph node metastasis, and poor prognosis) [22], miR222-3p (facilitates growth, metastasis, and invasion of a variety of malignant tumors), miR221-3p (predicting distant metastases and poor prognosis) [23], miR155-5p (closely related to the status of estrogen receptor (ER) and PGR) [24], and miR105-5p (potently inducing migration and proliferation in metastatic breast cancer (MBC) cells) [25]. Expression levels of miRNAs were analyzed using TaqMan™ MicroRNA assay probes and TaqMan™ Universal PCR Master Mix (Applied Biosystems, USA) in a 7900HT Fast Real-Time PCR System.

### Statistical methods

The main objective was to investigate the expression of five microRNAs in exosomes of BC patients and correlate their expression levels with clinical and pathological parameters and BC subtypes. The secondary objective was to test the association between the presence of CTC count at baseline and EmiR expression.

The statistical analyses were performed using R and SPSS 14.0 software (SPSS). To test if the microRNA expression was significantly different between patients with different clinicopathological parameters and BC subtypes, a non-parametric Wilcoxon signed-rank test was used when two groups were compared. To compare more than two groups, a non-parametric Kruskal-Wallis test was used instead. To test the correlation between number of CTCs and expression of EmiRs, Spearman’s rank correlation coefficient was used. Logistic binary regression and receiver-operating characteristics (ROC) curves were performed to test the sensibility and specificity of the Emir-21 (Ext1) and clinical biomarkers to identify MBC.

The presence of at least one CTC per 10 ml was considered a positive result according to the reported analytic detection limit of our assay [10]. Data are expressed as means or numbers (%). Two-tailed *p* < 0.05 values were considered statistically significant. Fisher test was calculated to assess the association between clinicopathological variables and the CTC status.

## Results

### Exosomal miRNA levels associated with LBC and MBC stages

After fully characterizing exosomes derived from cell lines (Additional file 1: Figure S1), we firstly compared the EmiR panel (21, 222, 221, 105, and 155) between 53 BC patients and 8 healthy donors to investigate the diagnostic role of these EmiRs and, secondly, between different disease stages: LBC and MBC to detect their prognostic implications.

**Fig. 1:**
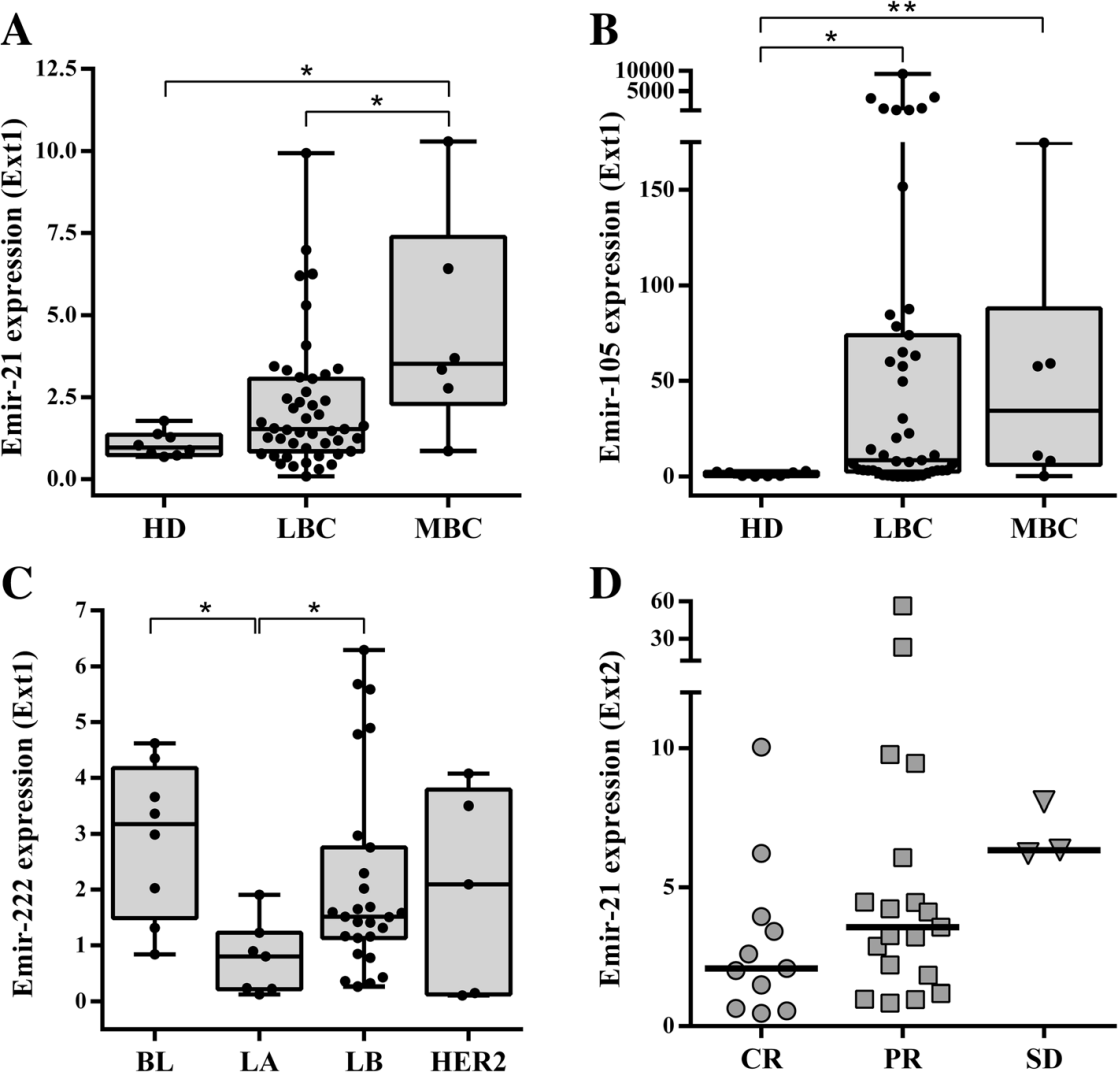
EmiR expression is associated with clinical stage, cancer subtype, and clinical response to neoadjuvant treatment. Expression of EmiR-21 (**a**) and EmiR-105 (**b**) compared between healthy donors (HD), non-metastatic (LBC), and metastatic (MBC) breast cancer patients. EmiR-222 (**c**) expression according to Perou’s classification in the LBC cohort, divided into four groups: basal-like (BL), luminal A (LA), luminal B (LB), and HER2. EmiR-21 (**d**) expression at Ext2 in patients with complete response (CR), partial response (PR), or stable disease (SD). Data are presented as a box and whiskers plot (min to max). *p > 0.05 and **p < 0.01. No significant comparisons are not represented

EmiR expression levels were statistically different between groups (healthy donors and LBC and MBC patients). Both EmiR-21 (*p* = 0.017) and EmiR-105 (*p* = 0.009) showed statistically significant differences between the three groups (Kruskal-Wallis test). Higher expression levels of EmiR-21 and EmiR-105 (*p* = 0.013 and *p =* 0.029 respectively) were found in MBC patients compared to healthy donors (Wilcoxon test). Furthermore, comparisons between LBC and MBC were found significantly different for EmiR-21 (*p* = 0.027) but not for EmiR-105 (*p* = 0.71). Emir-21 diagnostic potential of MBC was compared with the current clinical biomarkers carbohydrate antigen 19.9 and carcinoembryonic antigen by logistic binary regression. The Emir-21 was an independent diagnostic biomarker with a HR = 1.404 (95% CI = 1.028–1.918); (*p* = 0.033) (Additional file 1: Table S1) and the area under the curve (AUC) of the ROC curve was 0.777 (95% CI = 0.566–0.987) (*p* = 0.029) (Additional file 1: Figure S2). However, none of the clinical biomarkers could significantly identify MBC patients (Additional file 1: Table S1). Finally, EmiR-105 (*p* = 0.002), but not EmiR-21 (*p* = 0.09), was able to discriminate between healthy donors and LBC patients (Fig. 1a, b).

### Exosomal miRNAs and clinical characteristics in LBC patients under neoadjuvant chemotherapy

The association between expression levels of the EmiR panel at Ext1 and Ext2 and the clinical and pathological features are respectively summarized in Tables 1 and 2.

**Table 1:** Association between EmiR expression at Ext1 and clinicopathological characteristics in LBC patients

			Ext1									
			EmiR-21		EmiR-222		EmiR-221		EmiR-105		EmiR-155	
		*n*	Median	*p* value	Median	*p* value	Median	*p* value	Median	*p* value	Median	*p* value
Age (years)	< 50	27	1.518	0.983	1.506	0.966	1.161	0.667	11.199	0.897	1.901	0.302
	≥ 50	20	1.671		1.592		1.375		8.362		3.247	
Menopause	Pre	30	1.579	0.330	1.585	0.658	1.399	0.232	9.474	0.947	1.904	0.250
	Post	17	1.424		1.424		1.088		8.597		3.370	
T	T1–T2	27	1.265	0.039*	1.506	0.813	1.173	0.780	3.851	0.067	1.906	0.093
	T3–T4	20	1.787		1.556		1.379		22.184		4.073	
N	0	20	1.443	1.000	1.315	0.342	1.197	0.733	8.362	0.846	2.436	0.770
	1–3	23	1.543		1.584		1.088		30.239		3.019	
Estrogen receptor	Negative	13	1.615	0.812	2.992	0.161	1.221	0.812	49.757	0.140	2.776	0.981
	Positive	34	1.462		1.417		1.155		7.072		2.892	
Progesterone receptor	Negative	17	1.615	0.773	2.992	0.018*	1.597	0.465	49.757	0.116	2.776	0.947
	Positive	30	1.403		1.271		1.064		7.072		2.778	
HER2	Negative	36	1.531	0.466	1.465	0.880	1.149	0.563	8.362	0.744	3.117	0.421
	Positive	11	1.265		2.019		1.596		14.130		1.055	
KI67	< 20%	8	1.157	0.458	0.850	0.050	1.279	0.573	3.380	0.103	2.219	0.373
	≥ 20	37	1.518		1.584		1.161		14.130		3.019	
Perou’s classification	Basal-like	8	1.566	0.976	3.179	0.037*	1.191	0.986	39.998	0.306	4.127	0.495
	Luminal A	7	1.543		0.803		1.656		3.389		1.901	
	Luminal B	27	1.424		1.513		1.139		7.750		3.215	
	HER2neu	5	1.960		2.094		1.597		60.097		1.055	

Wilcoxon signed-rank test and Kruskal-Wallis test were used. *Abbreviations*: *CTCs* circulating tumor cells, *Ext1* basal extraction, *Ext2* extraction during neoadjuvant treatment, *T* tumor size, *N* lymph node status. *p > 0.05

**Table 2:** Association between EmiR expression at Ext2 and clinicopathological characteristics in LBC patients

			Ext2									
			EmiR-21		EmiR-222		EmiR-221		EmiR-105		EmiR-155	
		*n*	Median	*p* value	Median	*p* value	Median	*p* value	Median	*p* value	Median	*p* value
Age (years)	< 50	19	3.975	0.585	1.851	0.536	1.705	0.771	2.534	0.166	2.173	0.536
	≥ 50	14	3.033		2.100		1.718		11.182		1.401	
Menopause	Pre	20	4.159	0.094	2.250	0.461	1.890	0.338	3.916	0.224	2.177	0.768
	Post	13	2.192		1.551		1.422		10.603		1.507	
T	T1–T2	18	3.668	0.233	2.382	0.828	2.238	0.219	16.929	0.112	2.601	0.159
	T3–T4	15	3.246		1.851		1.286		3.484		1.819	
N	0	11	3.975	0.621	3.037	0.621	2.490	0.006*	21.588	0.445	1.344	0.928
	1–3	18	3.477		2.250		1.095		8.885		2.177	
Estrogen receptor	Negative	8	2.133	0.450	1.213	0.475	1.095	0.585	5.405	0.529	0.839	0.120
	Positive	25	3.933		2.649		2.000		11.760		2.173	
Progesterone receptor	Negative	11	2.192	0.541	1.338	0.760	1.787	0.541	7.327	0.789	3.224	0.849
	Positive	22	3.668		2.250		1.686		8.528		1.899	
HER2	Negative	27	3.933	0.031*	3.037	0.056	1.705	0.744	5.297	0.484	2.173	0.161
	Positive	6	0.857		0.536		41.874		46.810		0.639	
KI67	< 20%	6	4.996	0.427	1.464	0.283	0.321	0.176	3.691	0.963	1.355	0.191
	≥ 20	27	3.246		3.037		1.787		10.444		3.201	
Perou’s classification	Basal-like	5	2.192	0.655	1.338	0.666	1.286	0.666	3.484	0.510	0.983	0.232
	Luminal A	5	6.058		0.729		0.334		2.085		1.367	
	Luminal B	20	3.324		3.052		2.038		16.580		3.459	
	HER2neu	3	2.074		0.175		0.307		10.603		0.695	

Wilcoxon signed-rank test and Kruskal-Wallis test were used. *Abbreviations*: *CTCs* circulating tumor cells, *Ext2* extraction during neoadjuvant treatment, *T* tumor size, *N* lymph node status. *p > 0.05

At Ext1, 20 of 47 patients (42.55%) with larger tumors (III–IV) showed significantly higher EmiR-21 levels (*p* = 0.039) than those with smaller tumors.

According to hormone receptor expression at time of diagnosis, PGR-negative patients (17 of 47, 36.2%) showed a positive association with EmiR-222 expression (*p* = 0.017) at Ext1. Furthermore, 37 of 45 patients (82%) showed association between EmiR-222 at Ext1 and the proliferation marker Ki-67 (*p* = 0.050), that it was significantly associated with lower EmiR-21 levels at Ext2 (*p* = 0.030). With respect to HER2, an inverse significant association was found for EmiR-21 at Ext2 (*p* = 0.031) but not at Ext1 (*p* = 0.466). At the Ext2, we found lower levels of EmiR-221 Ext2 in lymph node-affected patients (*p* = 0.006).

### BC subtypes and EmiR profile

We correlated our five EmiR panel with the four major distinct molecular BC subtypes, according to Perou’s classification (Table 1) to address the clinical and relevant need of identifying subgroups. At Ext1, EmiR-222 was significantly associated with the different patient subgroups (*p* = 0.037): it was under-expressed in luminal A tumors vs. basal-like tumors (*p* = 0.004) and under-expressed in luminal A vs. luminal B (*p* = 0.015). However, non-significant differences were found between basal-like and luminal A/B, basal-like and HER2, or luminal A/B and HER2 (Fig. 1c).

### Predictive role of EmiRs

We analyzed the association of the EmiR panel at Ext2 with clinical response evaluated 3 months after treatment initiation. EmiR-21 did not show significant differences among partial response (PR), stable disease (SD), and complete response (CR) groups (*p* = 0.062) and neither when comparing CR patients vs. those with worse response to therapy (PR + SD) (*p* = 0.060) (Fig. 1d).

### Association between EmiR expression and CTC presence in LBC

We identified CTCs in 17 of 47 LBC patients (36.17%) at Ext1. Mean number of CTCs present was 1.23 cells per 10 ml of blood (range 0–10). At Ext2, CTCs were identified in 19 of 47 (40.43%) LBC patients, with a mean value of 1.60 cells per 10 ml (range 0–11). No significant differences were found between the presence of CTCs at Ext1 vs. Ext2 (*p* = 0.301 and *p* = 0.392 respectively). Correlation between CTC status and the clinical and pathological characteristics is shown in Additional file 1: Table S2.

**Fig. 2:**
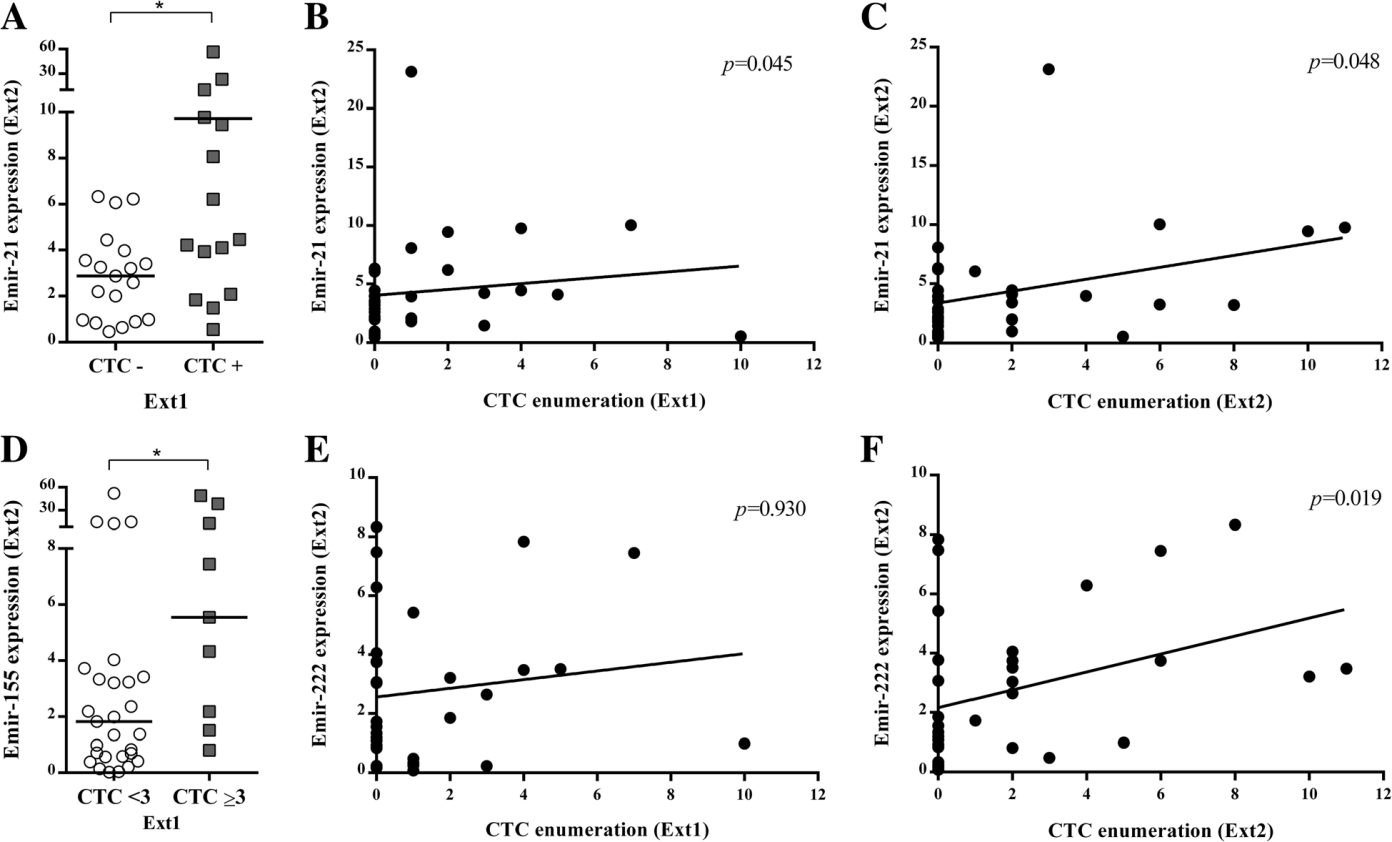
CTC detection correlates with EmiR expression. Comparison of EmiR-21 expression at Ext2 in patients with the presence or absence of CTCs at Ext1 (**a**), and correlation with the number of CTCs at Ext1 (**b**) and Ext2 (**c**). EmiR-155 expression comparison between patients with three or more CTCs per sample and less than three CTCs (**d**). Correlation of EmiR-222 expression and number of CTCs at Ext1 (**e**) and Ext2 (F). *p > 0.05

Moreover, EmiR-21, EmiR-222, and EmiR-155 showed a positive correlation with the presence and/or number of CTCs in LBC patients. In fact, higher levels of EmiR-21 at Ext2 were associated with the presence of CTCs at Ext1 (*p* = 0.032) (Fig. 2a). Besides, according to the number of CTCs, we observed higher EmiR-21 levels at Ext2 in those patients with higher number of CTCs at Ext1 (*p* = 0.045) and Ext2 (*p* = 0.048) (Fig. 2b, c). Furthermore, we observed a similar association between CTCs and EmiR-155 levels at Ext2. EmiR-155 was significantly overexpressed in the group of patients with ≥ 3 CTCs/10 ml at Ext1 (*p* = 0.039) (Fig. 2d). Finally, an association was found between higher levels of EmiR-222 and higher number of CTCs at Ext2 (*p* = 0.019) but not at Ext1 (Fig. 2f, e).

## Discussion

In the present study, we analyzed the role of five different exosomal miRNAs as a clinical tool to understand diagnostic, prognostic, and treatment response assessment.

Interestingly, one of the most important results observed in our study was the correlation between EmiR expression and disease stage. This way, EmiR-21 expression levels can distinguish localized from distant diseased patients. We also found differences in EmiR-105 levels between localized BC and healthy donors. These results might have important clinical implications for the correct identification of the disease and subsequent finest treatment choice. This could be particularly important in BC patients undergoing neoadjuvant chemotherapy, as it involves an advanced non-metastatic stage and includes a variety of clinical scenarios. A significative proportion of patients under neoadjuvant chemotherapy will end up developing metastatic disease, despite the administrated treatment. In addition, a percentage of them present distal disease at the time of diagnosis, which the current clinical methods are unable to detect. In consequence, these patients could be misdiagnosed and hence not appropriately treated. Therefore, the ability of LB to differentiate among localized disease, occult systemic disease, or not and healthy donors should be noted. A potential application would lay on the context of BC diagnostic, as despite the generally satisfactory results from mammograms, they have some important limitations, which can involve false negatives (i.e., greater proportion of cancers are detected after a negative mammogram). In fact, younger women (40–49 years) have lower mammographic sensitivity than older ones (≥ 50 years). We suggest that these limitations might be addressed by incorporating EmiR-21 and EmiR-105 analysis, in addition to mammogram tests. In this way, the LB could identify patients with metastatic disease, even those patients who are misdiagnosed as non-metastatic by the current clinical methods.

To assess the diagnostic ability of these EmiRs, we analyzed the correlation between EmiR levels in serum and different clinicopathological characteristics. miRNA-21 has been shown to be overexpressed in numerous types of tumor tissues [26], to be involved in cancer at almost all stages [27], and to be associated with proliferation and therefore growth. In the present study, serum EmiR-21 expression levels were positively correlated with tumor size, indicating that patients with higher serum EmiR-21 levels have larger tumors. Interestingly, we found lower levels of EmiR-221 during neoadjuvant treatment, in those patients with lymph node affection (N1-N3). The expression of miR-221 has been identified as a good prognostic biomarker in breast cancer tissues, and it is associated with ER positivity and lymph node negativity [28]. However, the miR-221 is associated with tamoxifen resistance in breast cancer cells [29]. In addition, Miller et al. [30] reported that miR-221/222 expressions were upregulated in endocrine therapy-resistant luminal-type breast cancer cells, which could explain our results. The association between EmiR expression levels, tumor size, and lymph node status suggests their potential beneficial use as diagnostic biomarkers to improve the stratification of metastasis risk in LBC.

We also observed lower levels of EmiR-21 in HER2-positive patients during neoadjuvant treatment with Trastuzumab (Ext2). Since EmiR-21 levels were not different between HER2-positive and HER2-negative patients at diagnosis time (Ext1), our results are in accordance with conclusions from similar studies [31, 32] suggesting that this low expression of EmiR-21 might be caused by the MAPK (ERK1/2) pathway blockage through HER2/neu. These data highlight the prognostic value of EmiR-21 to predict the treatment response of these patients to Trastuzumab.

Additionally, an inverse association was detected between EmiR-21 and Ki67 at Ext2. This correlation might predict the biological behavior of BC and its treatment response. Similar conclusions can be found in additional studies where the correlation among Ki67, HER2, and treatment was analyzed [33].

A correct BC diagnosis necessarily involves the identification of the different BC subtypes, what is crucial not only for risk stratification, but also for selecting the most appropriate patient treatment. Currently, identification of these BC subtypes depends on traditional pathological assessments with a limited number of biomarkers to define each subtype due to the technical impossibility of molecular profiling each tissue. However, despite the clinical relevance of these histopathological methods, the information provided is limited, with deficient genetic characterization of each patient and tissue. We observed associations between specific EmiRs and different BC subtypes. Our results, in agreement with the study of Sung EH et al. [34] in which miRNAs related to different BC subtypes and their target genes were identified, suggest that EmiRs could act as putative biomarkers valuable to improve classification and diagnosis of different BC subtypes. Therefore, identification of additional EmiRs will help to improve the genetic characterization of these tumor subtypes. Hence, we suggest that EmiRs, on further validation studies, as liquid biopsy biomarkers might become a non-invasive supplementary tool to the current BC classification methods. The use of these liquid biomarkers involves the possibility to obtain genetic information about the status of the disease on different temporal points, since BC is a dynamic disease that changes in the space and over time. These biological dynamics might be responsible for future relapses and resistance to treatment [35]. Therefore, the detection of changes in EmiRNA levels in biological fluids over time, might provide this information. Despite it was not the objective of our work, the EmiRNAs could be used to detect relapses in early stages and to detect early resistance to treatment.

Finally, we found a positive association between the presence of CTCs at baseline status and higher levels of EmiR-21, EmiR-155, and EmiR-222. As these miRNAs have been associated with tumor aggressiveness, promoting the proliferation and migration of tumor cells [22–24], higher levels of these EmiRs could explain more CTC dissemination.

## Conclusion

Currently used techniques are not sufficiently sensitive or specific so as to ensure a correct diagnosis of metastatic disease in breast cancer and certainly are unsatisfactory for therapeutic guidance. New approaches are needed to address this problem, requiring further research that involves novel markers and technologies to discover and validate improved biomarkers such as EmiRs. In this way, liquid biopsy based on serum EmiRs can become an additional clinical tool for improving BC diagnosis.

### Limitations of the study and interpretation

We would like to remark that our study included a single hospital with a limited cohort of patients; therefore, our results need further validation with a larger patient population. Inclusion of more patients, but also healthy donors, would improve the statistical power of our results. According with these limitations and as a result of the data of the EmiRNA profile established in this work, we are validating these results in different Spanish hospitals, using as central laboratory, the GENyO center.

On the other hand, the clinical utility of EmiRs should include reproducibility and analytic validity. These requirements involve the establishment of standard operating protocols (SOP) through multicenter clinical trials. These SOP not only should include the molecular analyses, but also include how the sample is collected, stored, and transported until the final molecular analyses. For LB to become a clinical reality instead of a simple proposal, the establishment and approval of these SOP by different laboratories and the performance of multicenter clinical trials is needed.

## Additional files

Additional file 1: **Figure S1.** Exosome characterization by TEM and Western blot. TEM images of exosomes derived from BC cell lines demonstrated that our methodology was successful in isolating exosomes, observing double-membrane vesicles with a diameter of ~ 150 nm (A). Furthermore, Western blot characterization showed positive expression of Hsp70 and CD9 exosomal proteins in these exosomes but negative expression of GM-130, which was present in the MCF-7 lysate positive control (B). **Figure S2.** ROC Curve of MBC identification by EmiR-21 (Ext1). Gray line represents EmiR-21 (Ext1) values for sensibility and specificity while black dotted line represents random predictor baseline. Light gray area represents EmiR-21 (Ext1) area under the curve (AUC) = 0.777. **Table S1.** Univariate logistic binary regression for MBC identification. Abbreviations: CA19.9, Carbohydrate Antigen 19.9; CEA, Carcinoembryonic Antigen; Ext1, basal extraction; HR, Hazard Ratio. **Table S2.** Association between CTC presence and clinicopathological features. Abbreviations: CTCs, circulating tumor cells; Ext1, basal extraction; Ext2, extraction during neoadjuvant treatment; T, tumor size; N, lymph node status. (ZIP 352 kb)

## Abbreviations

AUC: Area under the curve
BC: Breast cancer
cfNAs: Cell-free nucleic acids
CR: Complete response
CTCs: Circulating tumor cells
EmiRs: Exosomal microRNAs
ER: Estrogen receptor
Ext1: Diagnosis time
Ext2: After 4 cycles of doxorubicin/cyclophosphamide
LB: Liquid biopsy
LBC: Localized breast cancer
MBC: Metastatic breast cancer
N: Lymph node status
PGR: Progesterone receptor
PR: Partial response
ROC: Receiver-operating characteristics
SD: Stable disease
SOP: Standard operating protocols
T: Tumor size
