# Supplementary figures and images for: Exosomal miRNA profile as complementary tool in the diagnostic and prediction of treatment response in localized breast cancer under neoadjuvant chemotherapy
Source: Breast Cancer Res. 2019 Feb 6;21:21. doi: 10.1186/s13058-019-1109-0 (PMC6366103; doi:10.1186/s13058-019-1109-0)

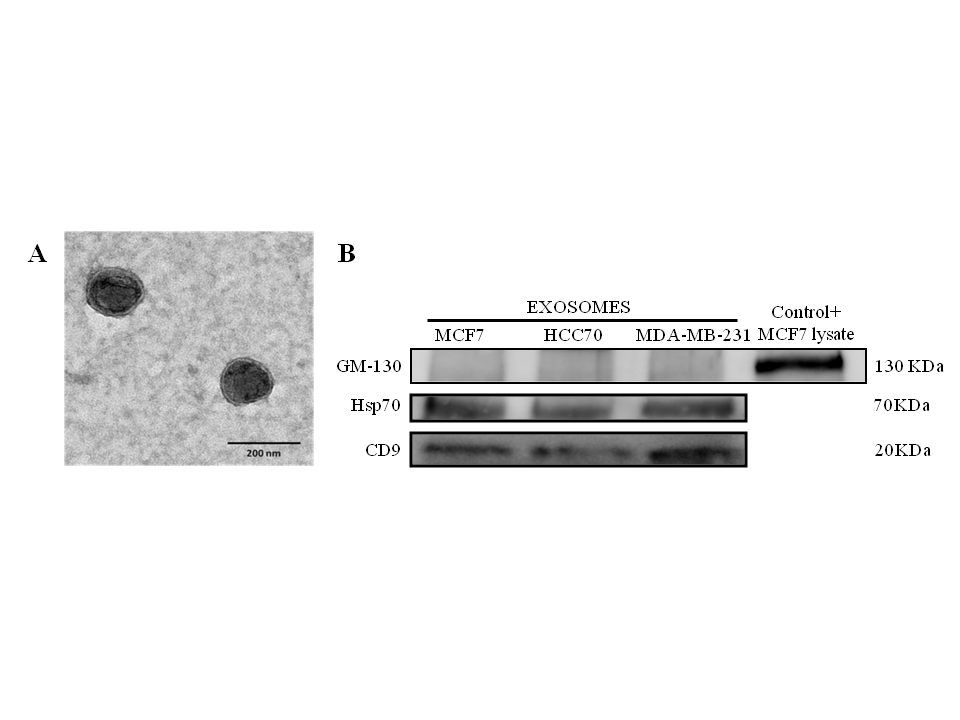

Supplement: Supplementary file 1 — Figure S1. Exosome characterization by TEM and Western blot. TEM images of exosomes derived from BC cell lines demonstrated that our methodology was successful in isolating exosomes, observing double-membrane vesicles with a diameter of ~ 150 nm (A). Furthermore, Western blot characterization showed positive expression of Hsp70 and CD9 exosomal proteins in these exosomes but negative expression of GM-130, which was present in the MCF-7 lysate positive control (B). Figure S2. ROC Curve of MBC identification by EmiR-21 (Ext1). Gray line represents EmiR-21 (Ext1) values for sensibility and specificity while black dotted line represents random predictor baseline. Light gray area represents EmiR-21 (Ext1) area under the curve (AUC) = 0.777. Table S1. Univariate logistic binary regression for MBC identification. Abbreviations: CA19.9, Carbohydrate Antigen 19.9; CEA, Carcinoembryonic Antigen; Ext1, basal extraction; HR, Hazard Ratio. Table S2. Association between CTC presence and clinicopathological features. Abbreviations: CTCs, circulating tumor cells; Ext1, basal extraction; Ext2, extraction during neoadjuvant treatment; T, tumor size; N, lymph node status. (ZIP 352 kb) [file 13058_2019_1109_MOESM1_ESM.zip › Supplementary F1.tif]

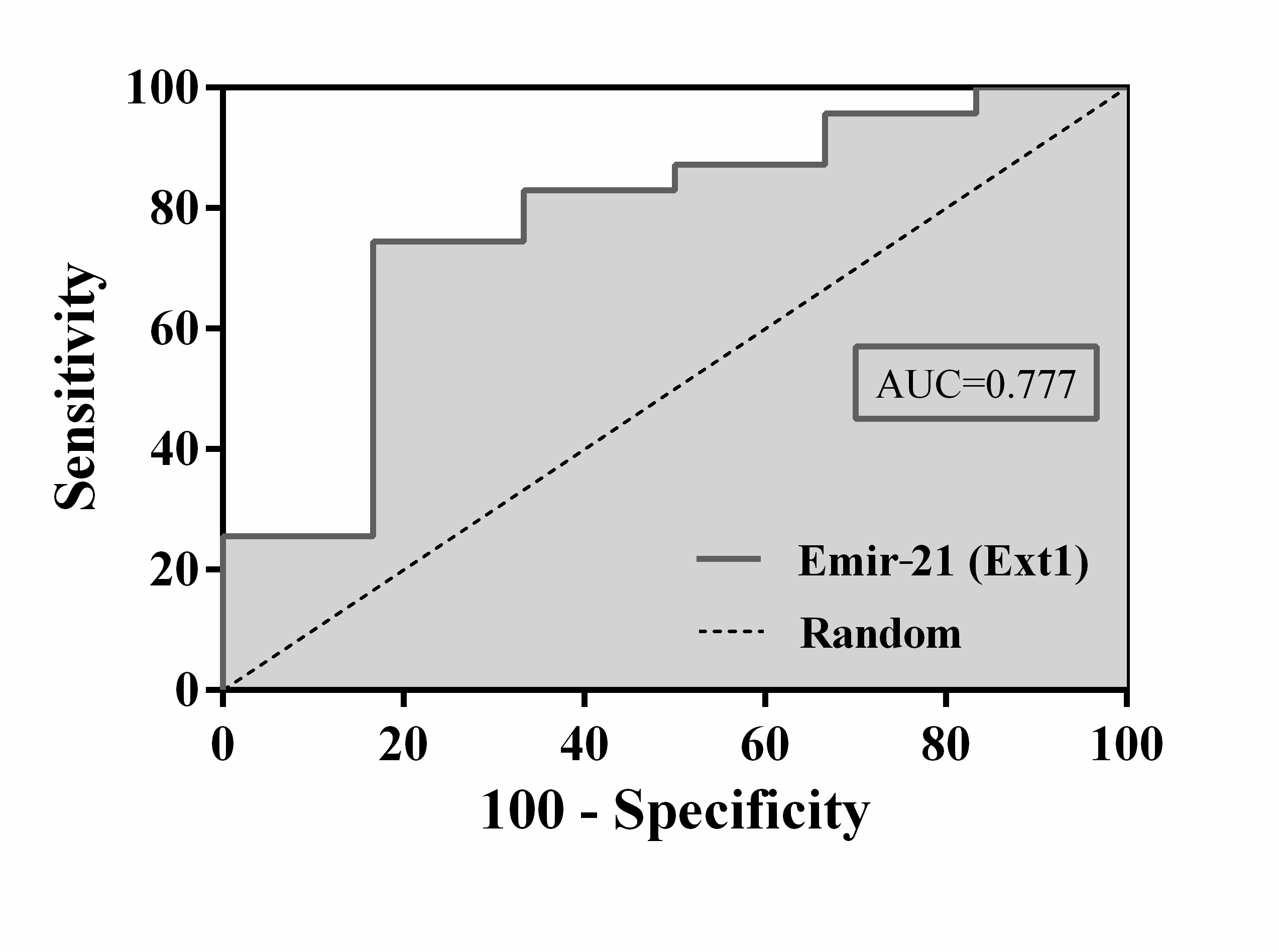

Supplement: Supplementary file 1 — Figure S1. Exosome characterization by TEM and Western blot. TEM images of exosomes derived from BC cell lines demonstrated that our methodology was successful in isolating exosomes, observing double-membrane vesicles with a diameter of ~ 150 nm (A). Furthermore, Western blot characterization showed positive expression of Hsp70 and CD9 exosomal proteins in these exosomes but negative expression of GM-130, which was present in the MCF-7 lysate positive control (B). Figure S2. ROC Curve of MBC identification by EmiR-21 (Ext1). Gray line represents EmiR-21 (Ext1) values for sensibility and specificity while black dotted line represents random predictor baseline. Light gray area represents EmiR-21 (Ext1) area under the curve (AUC) = 0.777. Table S1. Univariate logistic binary regression for MBC identification. Abbreviations: CA19.9, Carbohydrate Antigen 19.9; CEA, Carcinoembryonic Antigen; Ext1, basal extraction; HR, Hazard Ratio. Table S2. Association between CTC presence and clinicopathological features. Abbreviations: CTCs, circulating tumor cells; Ext1, basal extraction; Ext2, extraction during neoadjuvant treatment; T, tumor size; N, lymph node status. (ZIP 352 kb) [file 13058_2019_1109_MOESM1_ESM.zip › Supplementary F2.tif]
